# Addition and Cycloaddition Reactions of Phosphinyl- and Phosphonyl-2*H*-Azirines, Nitrosoalkenes and Azoalkenes

**DOI:** 10.3390/molecules14104098

**Published:** 2009-10-13

**Authors:** Américo Lemos

**Affiliations:** CIQA, Faculdade de Ciências e Tecnologia, Universidade do Algarve, Campus de Gambelas, 8005-137 Faro, Portugal; E-Mail: alemos@ualg.pt

**Keywords:** 2*H*-azirine, nitrosoalkene, azoalkene, phosphonyl, phosphinyl

## Abstract

An overview of the use of 2*H*-azirines, conjugated nitrosoalkenes and conjugated azoalkenes bearing phosphorus substituents in addition and cycloaddition reactions is presented, focused on strategies for the synthesis of aminophosphonate and aminophosphine oxide derivatives.

## 1. Introduction 

**Figure 1:**
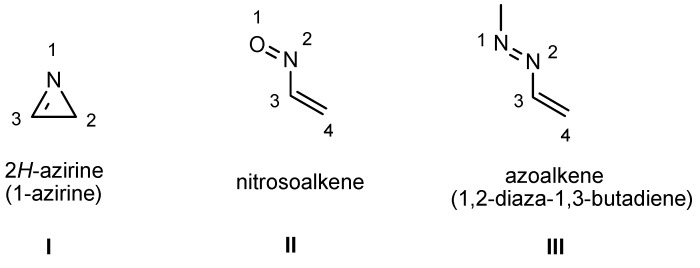
Name, structure and numbering of compounds.

Over recent years we and others have investigated the use of 2*H*-azirines, conjugated nitrosoalkenes and conjugated azoalkenes in nucleophilic addition and cycloaddition reactions. The structures **I**, **II** and **III** of these three classes are outlined in Figure 1.

A common feature of all three structures is that they possess a highly electrophilic carbon centre (C-3 in 2*H*-azirines, C-4 in conjugated nitroso- and azo-alkenes) that allows nucleophilic addition reactions to proceed very readily. In reactions of nucleophiles with 2*H*-azirines this often leads eventually to opening of the three membered ring. The electrophilic character of these structures also allows cycloaddition reactions, particularly those with nucleophilic olefins, to take place under very mild conditions. Such reactions have provided routes to a variety of novel heterocyclic structures which have proved to be very useful targets, not only due to their eventual biological and pharmacological properties, but especially to their wide and versatile use as synthetic intermediates or useful building blocks for the synthesis of amino acids, pyrroles, proline, indoles, pyrazines, and aza-sugars derivatives, amongst many other compounds [1,2,3,4,5,6,7,8,9,10,11].

Aminophosphonic and aminophosphinic acid derivatives can be considered as isosteres or surrogates of aminocarboxylic acids and they regulate various important biological functions [12,13,14,15,16]. In this context it is not surprising that organic chemists have been attracted to them and have paid particular attention to the synthesis of these types of compounds. The aim of this review is to illustrate the particular use of above reaction types of 2*H*-azirines, nitrosoalkenes and azoalkenes bearing phosphinyl or phosphonyl substituents, for the construction of alkyl *α*- and *β*-aminophosphonates and aminoalkylphosphine oxides.

## 2. 2*H*-Azirines

2*H*-Azirines are strained and activated imines. Their high reactivity makes them very useful synthetic intermediates for the synthesis of aziridines, amino acids, indoles, pyrazines, and other biologically active compounds through cycloaddition and nucleophilic addition reactions [3,5,6,8,9,17].

### 2.1. Synthesis

**Scheme 1:**
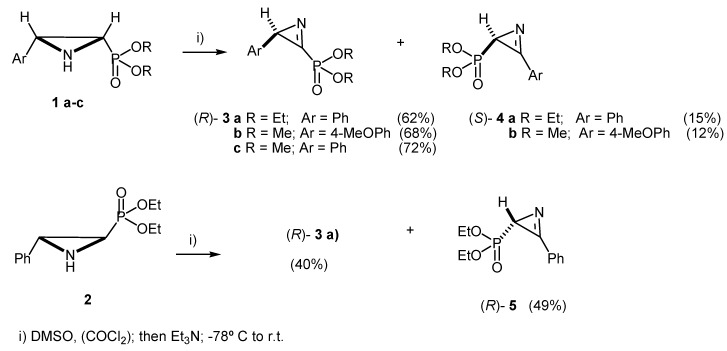
Synthesis of 2*H*-azirines by Swern oxidation.

Despite all these potential applications, 2*H*-azirines bearing phosphorus substituents have received comparatively little attention. Photocyclization of vinyl azides [18], reaction of phosphites with *β*-nitrostyrenes [19] and carbene addition to aromatic nitriles [20,21] constituted the earlier examples of 2*H*-azirines with a phosphinyl or phosphonyl functional group. Afterwards, a diverse methodology based on Swern oxidation of chiral aziridines **1** and **2**, produced regioisomeric mixtures of azirinyl phosphonates **3**-**5** (Scheme 1) [22,23].

**Scheme 2:**
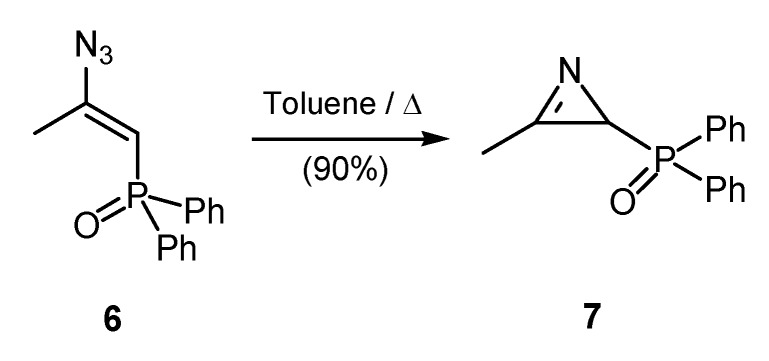
Thermolysis of vinyl azide **6**.

Thermolysis of vinyl azide **6** [24] allowed the isolation of diphenylphosphinyl 2*H*-azirine **7** in good yield (Scheme 2), but this strategy was not suitable for the preparation of enantiopure azirines.

**Scheme 3:**
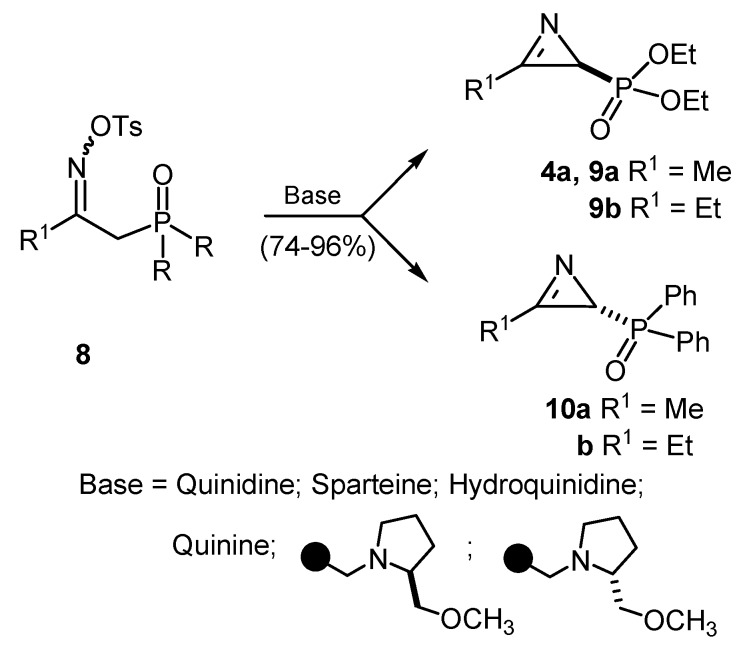
2*H*-Azirines by Neber reactions of *β*-keto tosyloximes.

The asymmetric synthesis of 2*H*-azirines bearing phosphinyl [24] and phosphonyl [25] substituents was disclosed by alkaloid mediated Neber reactions of *β*-keto tosyloximes. Similarly, the use of chiral polymer-supported bases [26] led to 2*H*-azirines **9** regioselectively and in high yields (Scheme 3).

**Scheme 4:**
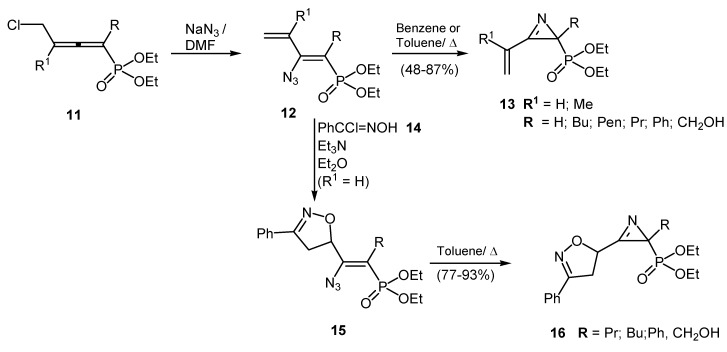
Synthesis of 3-vinyl- and 3-dihydroisoxazolyl-2*H*-azirines.

Another approach based on the treatment of phosphorylated allenes **11** with sodium azide was the basis of a convenient methodology for the synthesis of 3-vinyl- and 3-dihydroisoxazolyl-2*H*-azirines **13** and **16** (Scheme 4) [27,28].

### 2.2. Addition reactions

**Scheme 5:**
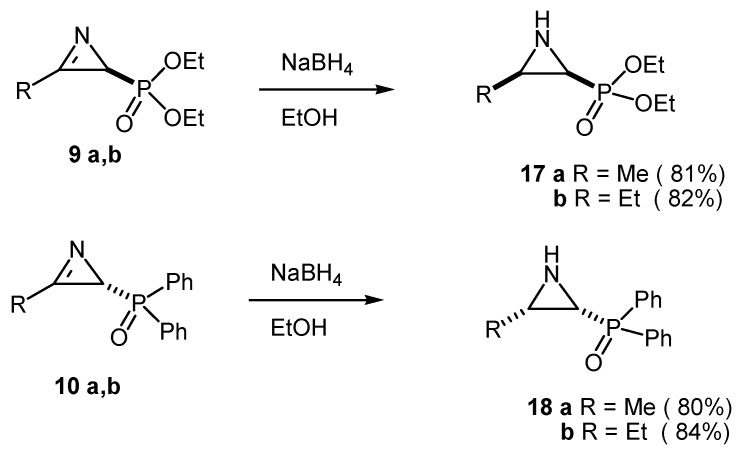
Hydride addition to 2*H*-azirines **9** and **10**.

One of the earliest reported reactions of 2*H*-azirines bearing phosphorus substituents was hydride addition [24,25]. The treatment of azirines **9, 10** with sodium borohydride in ethanol produced *cis*-aziridines exclusively (Scheme 5). The stereochemical assignment was based on the large coupling constant observed for the ring protons and further established by the transformation into enantiopure *cis-N*-(*p*-toluenesulfinyl)-aziridines by treatment with (-)-(*S*)-menthyl *p*-toluenesulfinate [24].

**Scheme 6:**
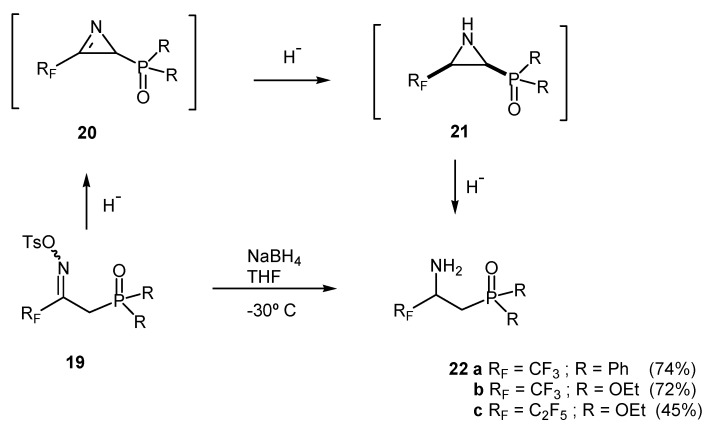
*β*-Amino-phosphine oxides and –phosphonates **22** from tosyloximes **19**.

For the synthesis of *β*-amino-phosphine oxide and –phosphonate derivatives **22** from tosyloximes **19**, a similar addition of hydride takes place with 3-fluoroalkyl-2*H*-azirines **20**–postulated as plausible intermediates – producing regioselectively *cis*-aziridines **21** which then lead to compounds **22** by ring opening [29] (Scheme 6).

**Scheme 7:**
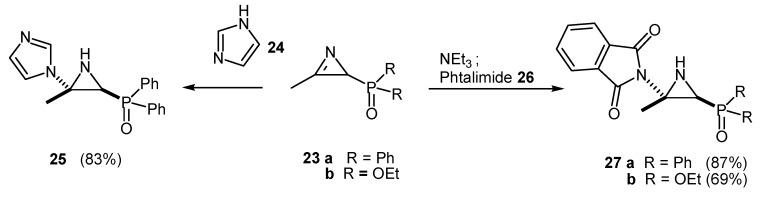
Addition of nitrogen heterocycles to 2*H*-azirines **23**.

**Scheme 8:**
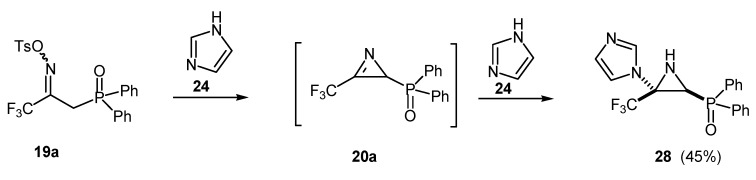
Imidazole mediated generation of 2*H*-azirine **20a** and nucleophilic addition.

Nitrogen heterocycles, in the presence or absence of base, add regioselectively to the azirine nucleus following the general pattern – the attack being from the less hindered face of the azirine - yielding functionalized aziridines [29,30] (Scheme 7 and Scheme 8). 

**Scheme 9:**
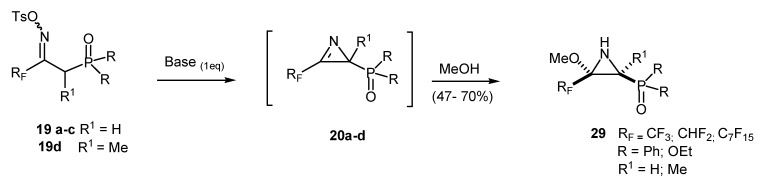
Methanol addition to 2*H*-azirines **20**.

**Scheme 10:**
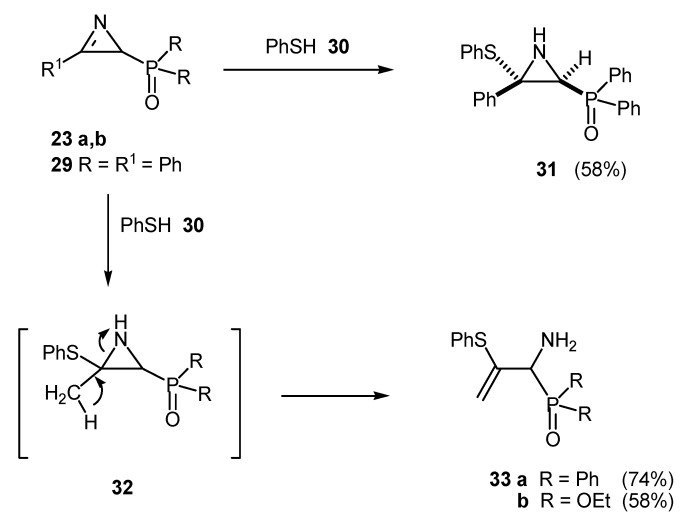
Benzenethiol addition to 2*H*-azirines **23** and **29**.

Oxygen [30] (Scheme 9) and sulfur [29] (Scheme 10) nucleophiles also add in a similar and regioselective mode, to 2*H*-azirines bearing phosphorus substituents. In the case of the reaction with benzenethiol, if a methyl group is present in the ring of the resulting aziridines, subsequent ring opening reaction leads to *a*-aminophospine oxide and –phosphonates **33**.

**Scheme 11:**
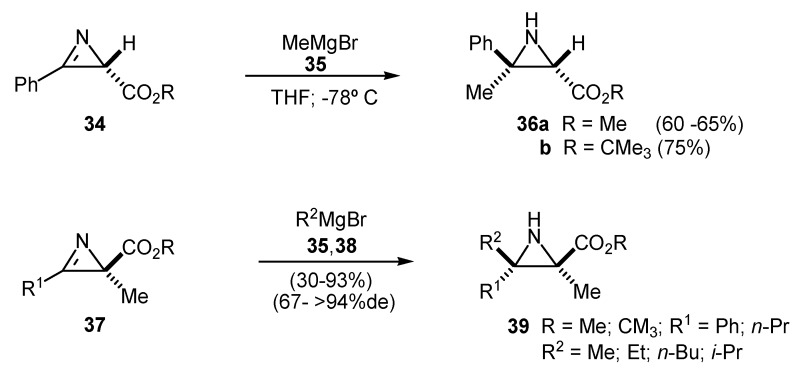
Addition of Grignard reagents to 2*H*-azirine-2-carboxylates.

The addition of Grignard reagents to 2-phosphinyl- and 2-phosphonyl-2*H*-azirines is less simple. Early reports with 2,3-diphenyl-2*H*-azirine revealed that the reaction followed the general pattern of addition of nucleophiles, i.e., the obtained aziridines arise from the attack at the less hindered face of the azirine [31]. These findings are in clear contrast with those obtained with alkyl 2*H*-azirine-2-carboxylates, in which the *syn* addition –to the more hindered face– is preferred (Scheme 11) [32,33]. These facts have been ascribed to a prechelating effect of the Grignard reagents with carboxylate substituents.

When the carboxylate group was replaced by a phosphoryl group, the reverse preference was observed, i.e., an exclusive attack at the least hindered side was encountered [30] (Table 1).

**Scheme 12:**
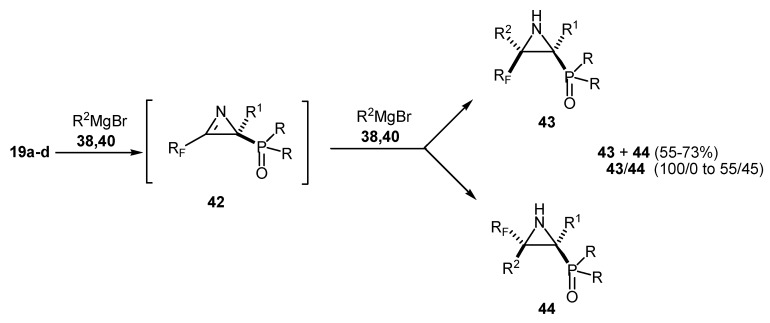
Generation of 2*H*-azirines **42** by Grignard reagents and nucleophilic addition.

This behaviour has been ascribed to the high exocyclic dihedral angle of the saturated carbon and to the presence of a bulky tetrahedral phosphorus group. But if a chelating substituent, such as alkylfluoromethyl or perfluoroalkylmethyl is present beside the phosphorus group, the former may play a major role in the mode of addition and in the reaction outcome, as demonstrated by the production of mixtures of *cis*/*trans* aziridines **43**/**44** [29] (Scheme 12).

**Scheme 13:**
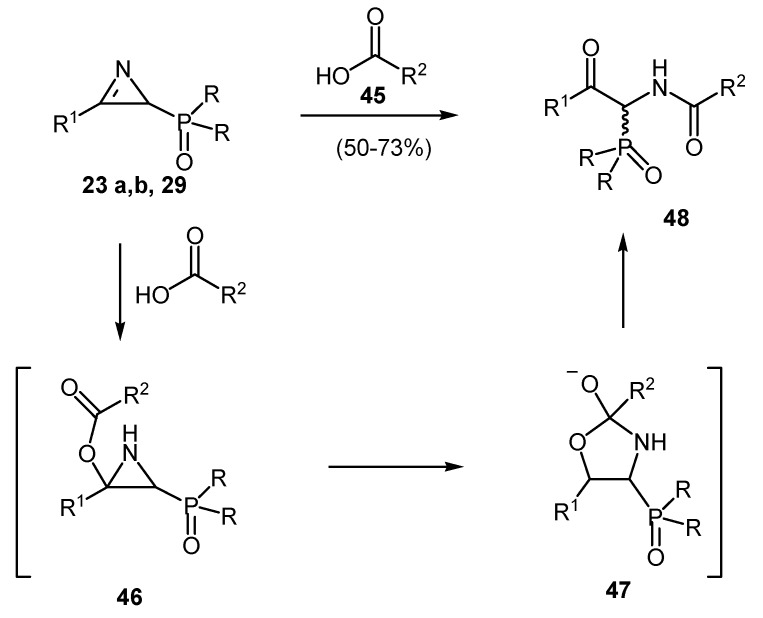
Ketamides **48** from 2*H*-azirines **23** and **29**.

Carboxylic acids [26], *N*-protected aminoacids and peptide residues [34] also add to the carbon nitrogen double bond of phosphinyl- and phosphonyl-2*H*-azirines. The concomitant ring opening leads to ketamides **48** (Scheme 13). 

Due to their ambident character, phosphinyl- and phosphonyl-2*H*-azirines also react as nucleophiles with carboxylic acid derivatives, such as acid chlorides, producing exclusively *trans*-aziridines **50** [35]. The scope of the reaction is not limited to simple chlorides since other functionalized acyl chlorides will react similarly in good overall yields. 

### 2.3. Cycloadditions

Simple alkyl- and aryl-2*H-*azirines, although being more reactive than acyclic imines, participate in Diels-Alder reactions only with highly reactive dienes, such as cyclopentadienones and 1,3-diphenylisobenzofuran in refluxing toluene [36], or with acyclic dienes and cyclopentadiene under Lewis acid catalysis [37,38]. 2*H-*Azirines with an alkoxy-, aryl-, amino-carbonyl [8,39] or heteroaromatic [40] substituent on the C=N bond are particularly good dienophiles in Diels–Alder reactions with a great variety of dienes, as a consequence of the conjugated effect of ring strain and extra activation by the electron-withdrawing group.

**Scheme 15:**
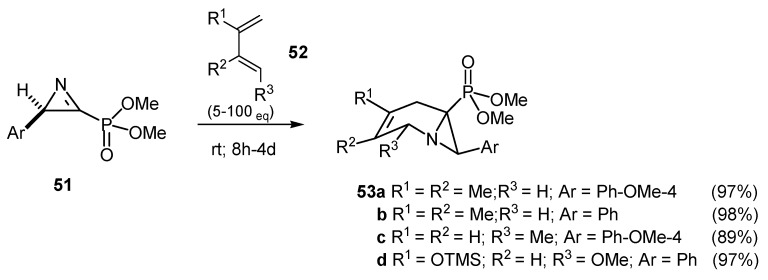
Cycloaddition reactions of 2*H*-azirine **51**.

Similarly enantiomerically enriched 2*H*-azirine-3-phosphonates **51** when stirred with 100 equiv of 2,3-dimethylbutadiene or *trans*-piperylene for 2-4 days at room temperature or with Danishefsky’s diene for 8 hours, afforded bicyclic aziridines **53** as single stereoisomers in good yields [23] (Scheme 15).

The stereochemistry of cycloadducts **53** was consistent with exclusive addition of the diene to the less hindered face of the azirine **51**. The longer reaction times, when compared with 2*H*-azirine-3-carboxylates, may suggest that 2*H*-azirine-3-phosphonates are less reactive than carboxylates.

**Scheme 16:**
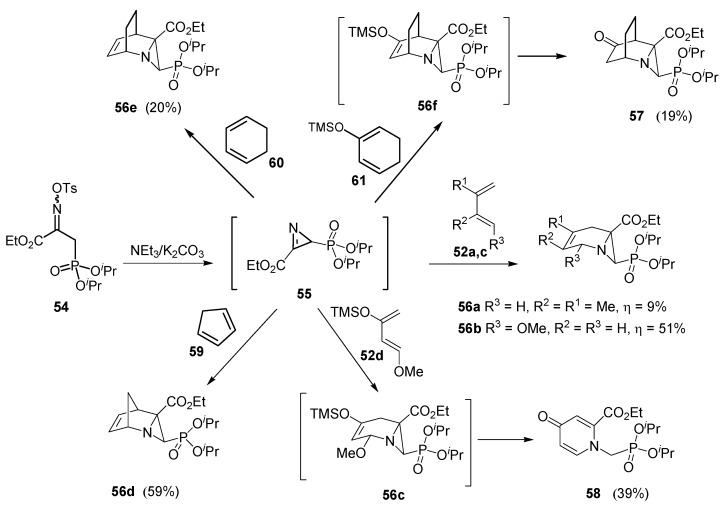
Cycloaddition reactions of 2*H*-azirine **55** with nucleophilic dienes.

Recently an azirine bearing both ethoxycarbonyl and phosphonate groups, was generated *in situ* and intercepted with a number of nucleophilic dienes [41]. With open chain dienes bicyclic functionalized six-membered ring fused aziridines were produced; although cyclic dienes afforded tryciclic structures. The presence of a trimethylsilyloxy group at the conjugated system, induced hydrolysis of cycloadduct **56f** and **56c** to **57** and **58** respectively (Scheme 16). 

The products were isolated as single isomers, presumed to be formed by *endo* selective processes, as clearly indicated by the low field resonance of H-3 in the tricyclic structure **56d**, attributed to the anisotropy of the backside double bond over H-3, due to constrain of the tricyclic structure [42]. To the poor stability of azirine **55** was ascribed the low to moderate yields of cycloadducts (Scheme 16).

## 3. Nitroso- and Azo-alkenes

Nitrosoalkenes and azoalkenes, used either as Michael-type acceptors in conjugate 1,4-additions, or as heterodienes in cycloaddition reactions with a range of nucleophiles, alkenes and heterocycles, have proved to be invaluable tools for the synthetic organic chemists.

### 3.1. Synthesis or generation

**Scheme 17:**
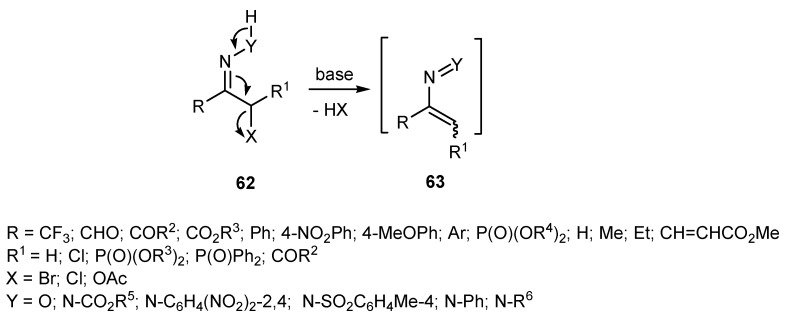
General method for the obtention of nitroso- and azo-alkenes.

Electron-deficient nitrosoalkenes are, generally, very unstable species and for this reason, they are usually generated and intercepted *in situ*. Depending on the substituents, azoalkenes are sometimes stable enough to be isolated. Anyway, being isolated or generated “*in situ*”, the most common, general and broad scope method for the obtention of nitroso- and azo-alkenes is the base induced 1,4-dehydro elimination from oximes and hydrazones bearing a suitable halogen or ester leaving group at the *α*-position [1,2,4,7,10,11] (Scheme 17).

### 3.2. Reactions with nucleophiles

Although in reactions involving nitroso- and azo-alkenes generated and intercepted *in situ*, the distinction between nucleophilic substitution of the original *α*-halogenated oxime or hydrazone and 1,4-conjugate (or Michael type) addition, is sometimes an intricate decision, the following reactions are thought to proceed *via* this latter process.

The primary literature reference to azoalkenes bearing phosphorus substituents [43] reported their use at the synthesis of 1-aminopyrroles substituted with a phosphine oxide or phosphonate group in the 3-position.

**Scheme 18:**
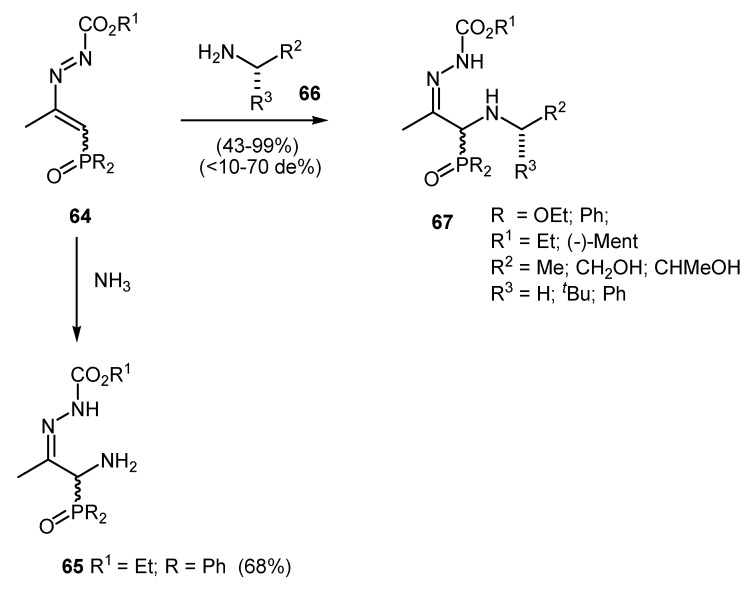
Nucleophilic addition of ammonia, aminoesters and aminoalcohols to azoalkene **64**.

Achiral and chiral phosphinyl- and phosphonyl-1,2-diaza-1,3-butadienes obtained from hydrazonoalkyl-phosphine oxides and –phosphonates, were reported to add ammonia, aminoesters and aminoalcohols giving functionalized *a*-amino-phosphine oxides and –phosphonates [44,45] (Scheme 18). Very low diastereoselection with optically active amines was encountered - the adducts were isolated as nonseparable diastereoisomeric mixtures. Better diastereoselection was found when the bulky (*S*)-*tert*-leucinol substituent was used.

**Scheme 19:**
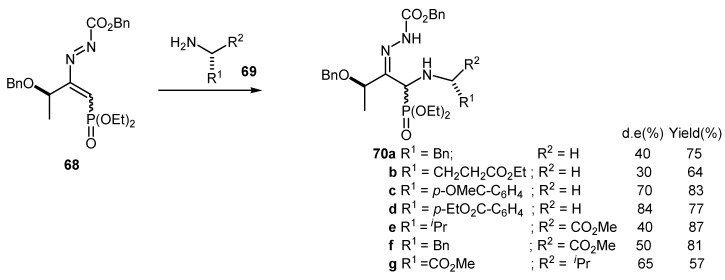
Addition of amino esters to optically active azoalkene **68**.

The diastereoselective addition of optically active amino esters gave slightly improved results when an optically active group was introduced at position 3 of the azoalkene. Thus the azovinylphosphonate **68** produced hydrazono derivatives **70** in good yields but moderate diastereoselectivity [46] (Scheme 19).

**Scheme 20:**
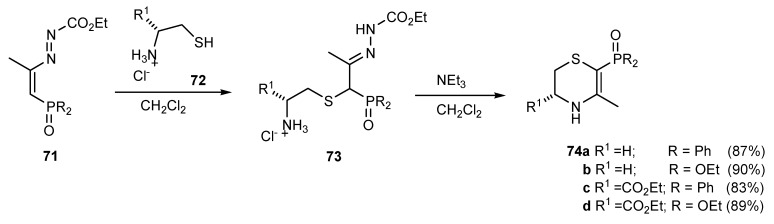
1,4-Addtion of 1,2-aminothiols **72** to nitrosoalkenes **71**.

**Scheme 21:**
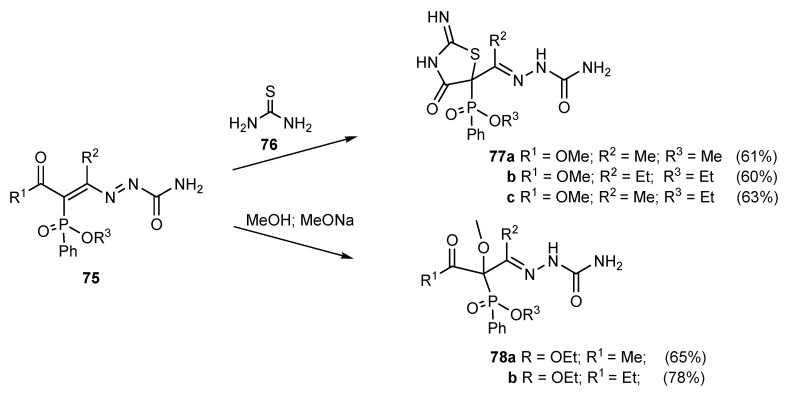
Addition of thiourea and methanol to 1,2-diaza-1,3-butadienes **75**.

**Scheme 22:**
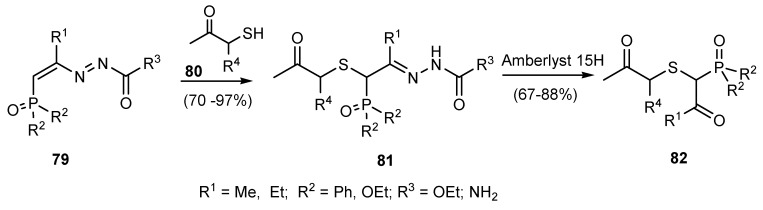
Addition of 3-mercapto-2-ketones **80** to azoalkenes **79**.

Amino- and alkoxy-carbonyl 1,2-diaza-1,3-dienes underwent 1,4-addtion with sulfur nucleophiles such as 1,2-aminothiols [47] (Scheme 20), thiourea [48] (Scheme 21) and 3-mercapto-2-ketones [49] (Scheme 22) producing either functionalized hydrazones or ensuing cyclised heterocycles.

Similarly, hydrazones **78a,b** arised from the 1,4-addition of oxygen nucleophiles to 1,2-diaza-1,3-dienes [48] (Scheme 21).

**Scheme 23:**
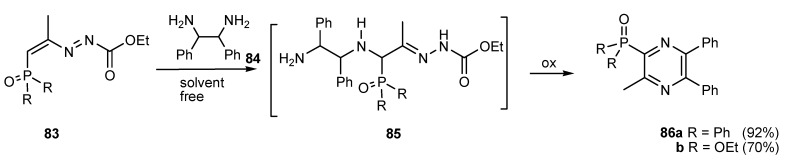
Addition of 1,2-diamines to phosphinyl- and phosphonyl-azoalkenes **83**.

Under solvent free or solid-phase conditions, a Michael type addition of 1,2-diamines to phosphinyl- and phosphonyl-azoalkenes was observed, furnishing substituted hydrazones which subsequently were cyclised into pyridazines [50] (Scheme 23).

**Scheme 24:**
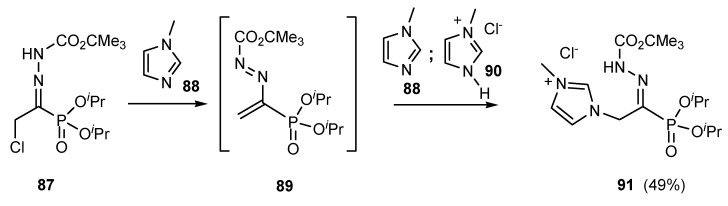
*N*-methylimidazole generation of azoalkene **89** and nucleophilic addition.

The nitrogen heteroaromatic *N*-methylimidazole was reported to promote dehydrohalogenation of hydrazone **87** and to add regioselectively to the azo-alkene **89**, conducting to functionalized *α*-hydrazonophosphonate **91** [51] (Scheme 24). 

**Scheme 25:**
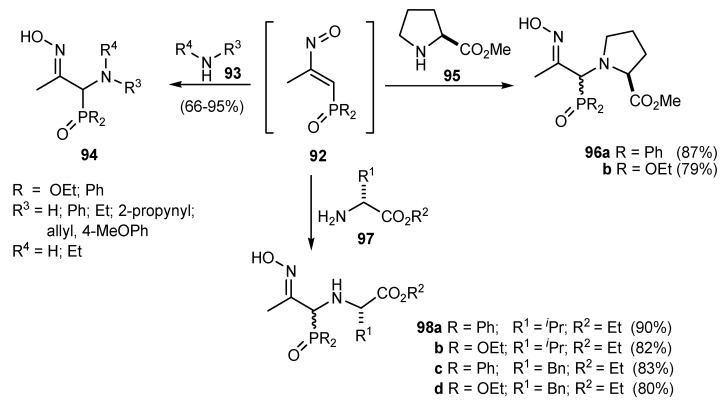
Nitrosoalkenes **92** as Michael acceptors.

Several examples of nucleophilic conjugate addition to nitrosoalkenes are reported in the literature [4,10,52,53,54], whereas reports of related reactions of phosphinyl- and phosphonyl-nitrosoalkenes are scarcely described. Nitrososalkenes **92**, generated “*in situ*” from the corresponding α-bromooxime, were reported to act as Michael acceptors towards ammonia, amines and optically active amino esters, affording *α*-amino phosphine oxides and *α*-amino phosphonates [55] (Scheme 25). The reactions, although being regioselective, lacked stereoselectivity with optically active compounds. 

There are various works reporting reactions of conjugated nitrosoalkenes with enamines [52,56,57,58,59,60] affording 1,2-oxazines in good to excellent yields *via* postulated Diels-Alder reactions with inverse electron demand. A more recent work [61] using density functional theory (DFT) studies, showed a polar character of the Diels–Alder reaction of nitrosoalkenes with enamines, being the two σ bonds formed in these polar cycloaddition reactions in a highly asynchronous concerted process. On the other hand reactions of azoalkenes with enamines are solvent and structure – of enamine and azoalkene - dependent [1,62,63,64,65,66,67,68,69], proceeding either *via* [4+2] cycloaddition or conjugate addition.

**Scheme 26:**
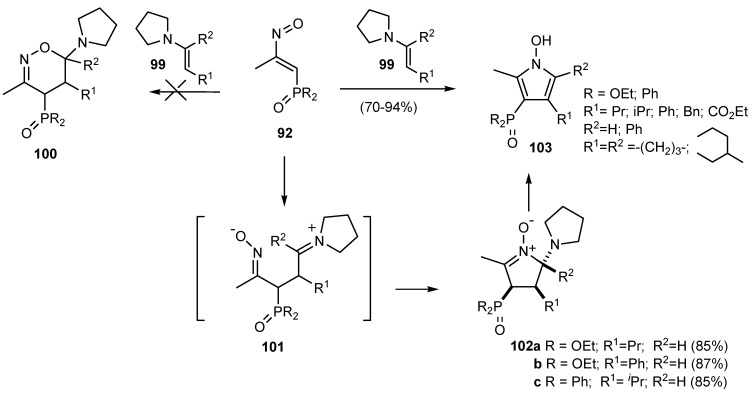
Reaction of nitrosoalkenes **92** with enamines **99**.

In this context, Palacios and co-workers [70] found that the reactions of nitrosoalkenes, bearing a phosphinyl or phosphonyl substituent at the terminal carbon, with enamines **99** did not proceed by [4+2] cycloaddition reactions producing the expected 1,2-oxazines, but *N*-hydroxypyrroles were instead isolated, presumably by a mechanism involving an initial conjugate addition of the enamine followed by a formal [3+2] dipolar cycloaddition (Scheme 26).

### 3.3. Cycloadditions

Cycloaddition reactions of nitroso- and azo-alkenes with electron rich carbon-carbon double bonds and heterocycles are important and powerful synthetic tools, as demonstrated by the enormous impetus of the chemistry of these compounds over the last decades. 

**Scheme 27:**
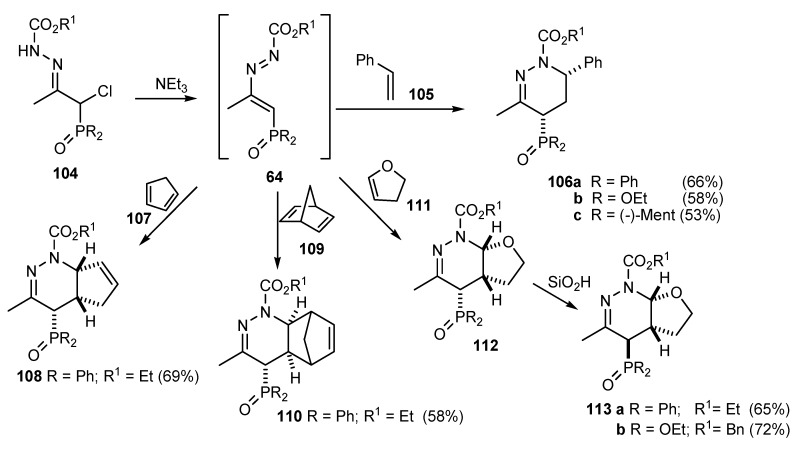
Cycloaddition reactions of azoalkenes **64**.

Azoalkenes with a phosphinyl or phosphonyl substituent at the 4-position were generated by triethylamine induced dehydrohalogenation of chlorohydrazones [71]. Its interception with acyclic— styrene and cyclic—cyclopentadiene and norbornadiene alkenes and di-hydrofuran gave regio- and stereo-selectivelly *endo* cycloadducts, except in the case of the strained norbornadiene, which produced an *exo* cycloadduct (Scheme 27).

Attempts to bring about some degree of diastereoselectivity to the reaction, induced by the use of an optically active (*-*)-menthyl ester, were unsuccessful, since a diasteroisomeric ratio of 1:1 was found.

**Scheme 28:**
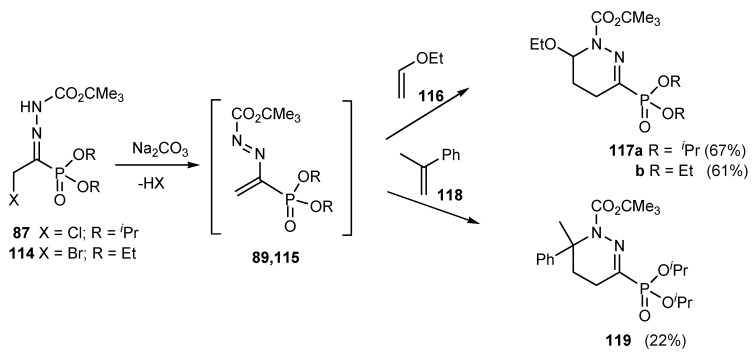
Cycloaddition reactions of azoalkenes **89** and **115** with electron rich olefins.

Cycloadducts were also obtained in regioselective fashion in reactions of azoalkenes bearing a phosphonate substituent at the 3-position with electron rich olefins [51] (Scheme 28). 

Reactions with electron rich heterocycles such furan and dihydrofuran gave rise to the formation of the corresponding cycloadducts. With pyrrole and indole open chain hydrazones were isolated (Table 2), assumed to be the result of rearomatization of the primarily formed cycloadducts [72]. The yields were, in general, lower than those obtained in similar reactions with azoalkenes bearing the same alkoxycarbonylazo substituent, but having an ethoxycarbonyl group at the 3-position [73], pointing out that eventually the ethoxycarbonyl group may play a more effective role than the phosphonate moiety.

**Scheme 29:**
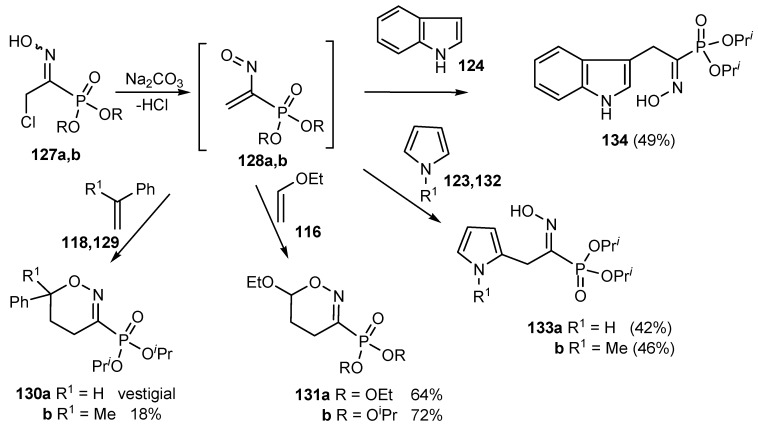
Cycloaddition reactions of nitrosovinylphosphonates **128**.

Nitrosovinylphosphonates generated from the corresponding chlorooximes were intercepted by electron-rich alkenes and heterocycles [74]. The yields, although not optimised, were fair and the reactions were found to be completely regioselective: no other isomers were detected or isolated from the reaction medium (Scheme 29). 

## 4. Concluding Remarks

Synthetic applications of 2*H*-azirines, nitroso- and azo-alkenes bearing phosphinyl or phosphonyl substituents, emphasised towards the synthesis of *α*- and *β*-amino-phosphonates and –phosphine oxides have been addressed in this review. Established methods of aziridine ring opening [17,75] and reductive transformations at the C=N bond [76-78] will further broaden the scope and importance of these strategies that surely will be further developed and attract the attention and interest of synthetic chemists.

## Figures and Tables

**Scheme 14:**
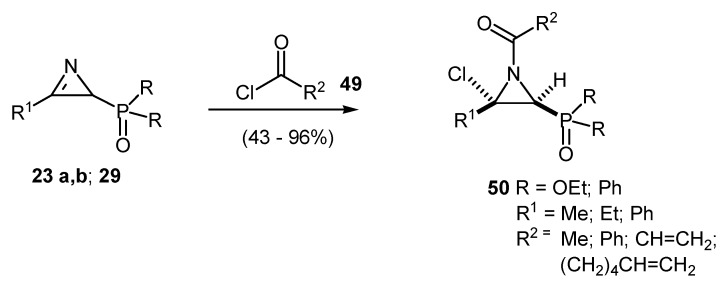
Reactions of acyl chlorides with phosphinyl- and phosphonyl-2*H*-azirines.

**Table 1:**
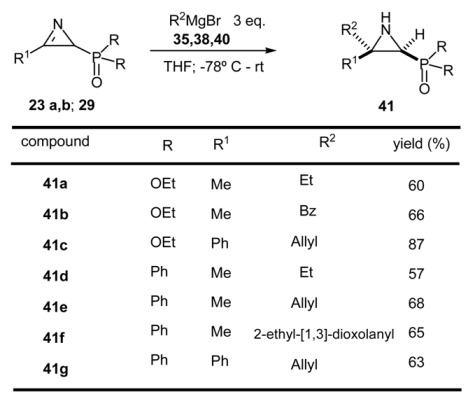
Addition of Grignard reagents.

**Table 2:**
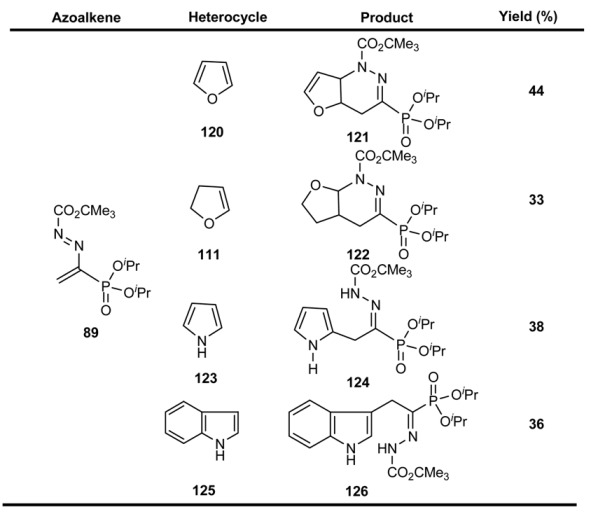
Reactions of azoalkene **89** with electron-rich heterocycles.
